# Vaginal Examination Simulation Using Citrus Fruit to Simulate Cervical Dilation and Effacement

**DOI:** 10.7759/cureus.314

**Published:** 2015-09-01

**Authors:** Kathleen L Shea, Edward J. Rovera

**Affiliations:** 1 School of Nursing, San Francisco State University

**Keywords:** nursing education, nursing simulation, intrapartum vaginal examination, pre-licensure nursing education, cervical dilation, cervical effacement

## Abstract

This technical report describes the creation and use of a cervical dilation and effacement model in a pre-licensure nursing course in reproductive health. Vaginal examination is typically taught in reproductive health courses; however, nursing students do not always have sufficient opportunity to practice on actual patients. This low-cost task-training model provides undergraduate nursing students the opportunity to experience performing a vaginal examination to assess for cervical dilation and effacement during the labor process.

## Introduction

As simulation education and training become increasingly prevalent in nursing education, the costs of capital equipment and supplies grows. This increase in expenditures taxes the resources of many pre-licensure nursing programs [1]. In addition, the limited number of clinical sites in Labor and Delivery in most geographic areas makes simulation an attractive curricular addition to supplying the required number of clinical hours for students [2-3].

Maternity textbooks used in pre-licensure nursing education normally describe the technique for performing a vaginal examination to assess cervical dilation, effacement, station, and presentation, but few students get the opportunity perform a vaginal examination during their maternity nursing practicum [4]. Because of this lack of direct experience in baccalaureate nursing education, the authors sought an inexpensive cervical model to improve the novice nursing student’s understanding of the intrapartum vaginal examination.  

A review of the simulation literature in obstetrics yielded few published descriptions of the creation and use of manual vaginal examination task trainers and only two that offered a low-cost vaginal model for practicing manual vaginal examinations. Nitsche and Brost developed an artificial vaginal vault made of PVC pipe and frozen beef to simulate the cervix [5]. Pratinidhi, et al. built a wooden device with a rotating drum that will present different rubber cervical models to a hole through which medical students insert their fingers in a simulated manual vaginal examination [6].

Given that few low-cost cervical task trainers exist, the authors felt the creation and use of a cervical dilation and effacement model may prove of value when providing students with training in this area of nursing. This report presents the creation and use of a low-cost cervical dilation and effacement model using citrus fruit and tube socks.

## Technical report

To create a simple model of a cervix in the midst of dilation and effacement, a clinical instructor suggested carving out a small circle of the rind of an orange -- approximately 5 cm in diameter. This could be shown to the students, and they would have the opportunity to feel the edges. The circle could then be enlarged and the edge trimmed down to represent effacement. Again, this model could be shown to the students, and they would have the opportunity to feel the now flatter edge of the circle. Clinical faculty with experience in labor and delivery tested this method and determined that the orange did represent the tactile qualities the authors were seeking.

After experimenting with various sizes of citrus fruit and considering several ways to simulate the vaginal vault, tube socks were selected. They provided a supple material that could be shaped by folding the sock back while covering the simulated cervix sufficiently to require the student to feel the dilation and effacement. An exercise was developed for the purpose of introducing the manual vaginal examination to pre-licensure nursing students in the third semester of a five-semester program. The equipment and supplies needed for the exercise are listed below.

### Equipment and supplies

-Six to ten oranges and grapefruit

-Six to ten tube socks

-Centimeter ruler to set cervical diameters

-Scalpel, utility knife, or other sharp cutting tool

-Felt tip marking pen

-Blue painter’s tape

-Labels for the completed models

-Student recording sheets (Table 1)

**Table 1:** Student Recording Sheet

Simulated Vaginal Exam	
Name: ________________________________	Date: ____________________________________

	Dilation in Centimeters	Effacement as a Percentage
A	__________________________________	__________________________________
B	__________________________________	__________________________________
C	__________________________________	__________________________________
D	__________________________________	__________________________________
E	__________________________________	__________________________________
F	__________________________________	__________________________________
G	__________________________________	__________________________________
H	__________________________________	__________________________________
	Example	Example
#	5 cm	60%

-Gloves -- non-sterile or sterile, depending upon the fidelity you are seeking

-Water-soluble lubricant -- non-sterile or sterile, depending upon the fidelity you are seeking

-Two or three small prizes such as candy, pens or pencils

To assemble the models, follow the model assembly steps below. The authors found that two people working together can create six to eight models in approximately one hour.

### Model assembly

**Figure 1:**
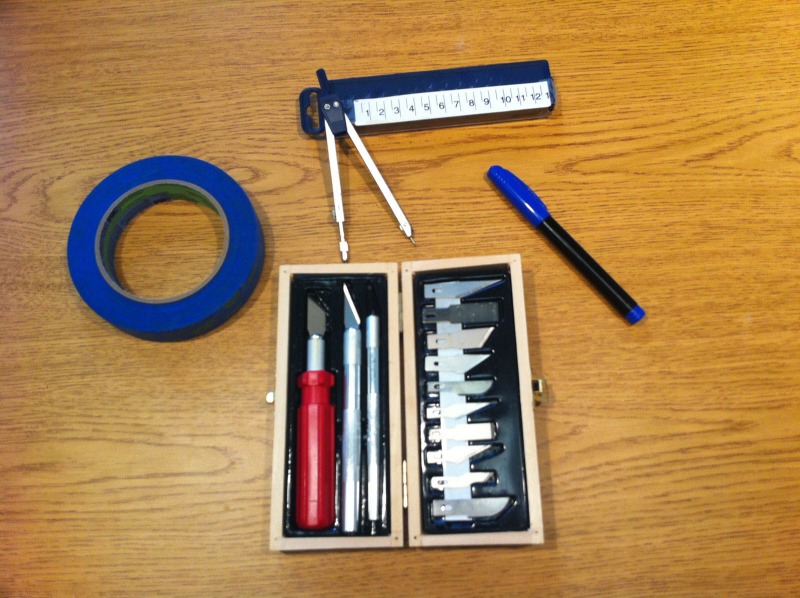
Tools needed to create models

-Gather the required tools (Figure 1).

-Mark the fruit with desired diameters and effacements for each model. TIP: Use navel oranges and thick rind grapefruit for models with a low percentage of effacement.

-Remove the pencil tip from the draftsman’s compass.

**Figure 2:**
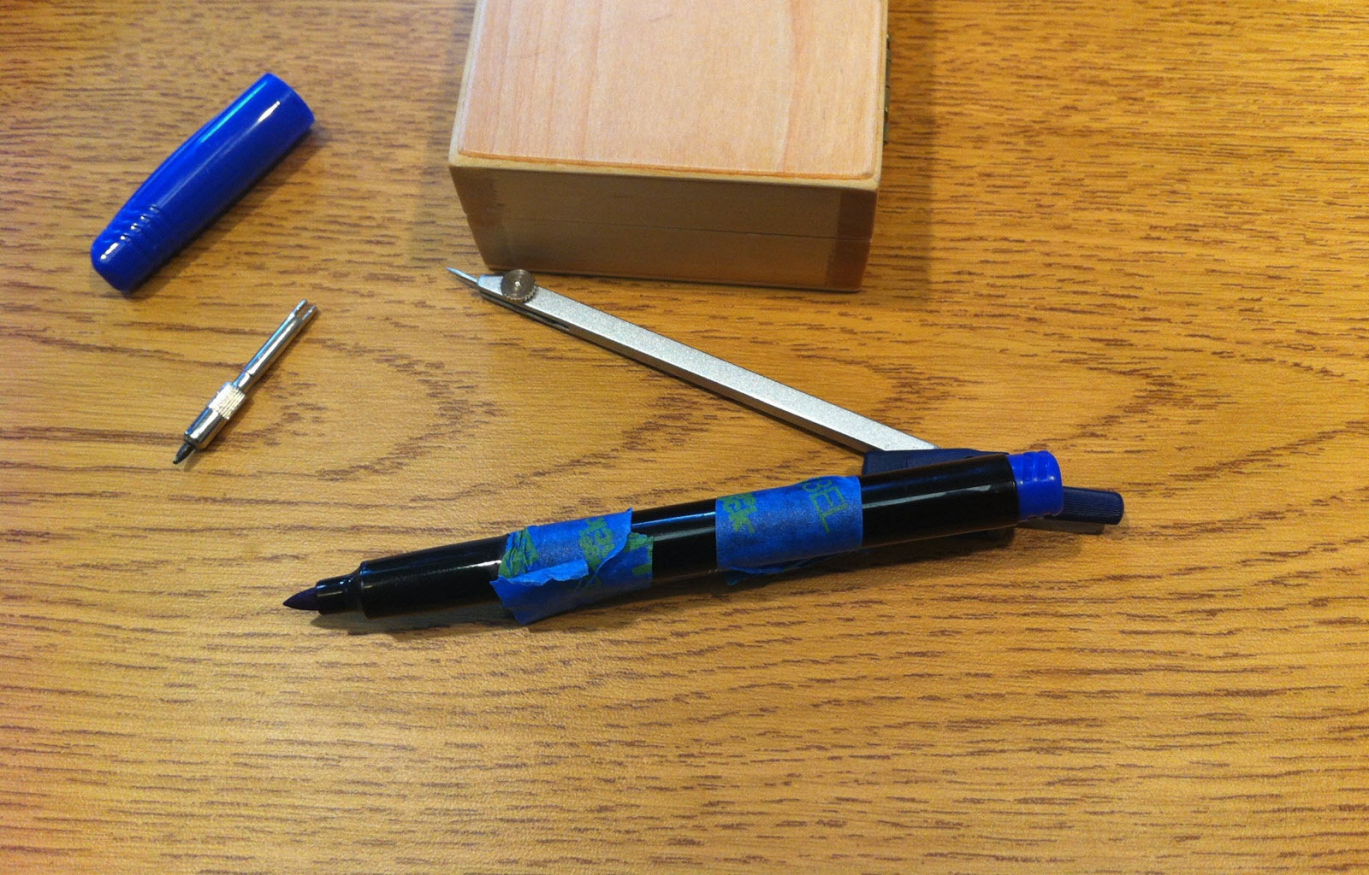
Felt-tipped pen secured to draftsman's compass

-Tape a fine-tip felt marking pen to the compass so the tip of the pen extends approximately as far as the pencil tip had extended (Figure 2).

-Use the centimeter rule to set the compass to the desired cervical radius.

-Press the compass needle point into the fruit at the center of desired cervix.

-Touch the pen tip against the fruit.

-Without lifting the pen tip off the fruit, gently rotate the fruit under the compass until you have created a complete circle on the surface of the fruit.

**Figure 3:**
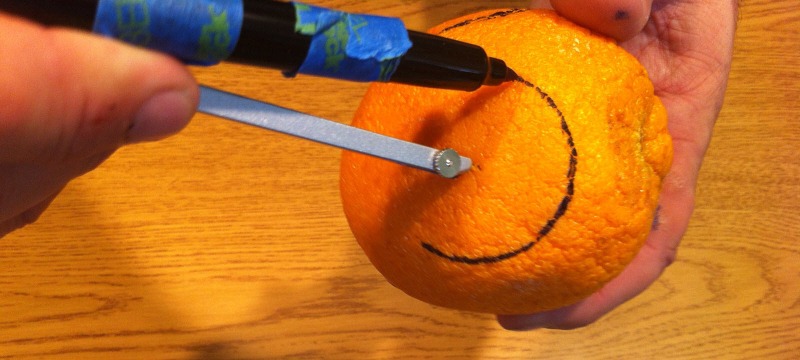
Creating cervical diameter on the citrus fruit Remember to place the pen tip against the skin of the fruit and turn the fruit. This reduces variation in the radius significantly over attempting to turn the compass with the fruit stationary.

NOTE: Rotating the fruit under the compass rather than turning the compass helps to avoid undesired variations in the radius (Figure 3).

-Using your cutting tool, gently cut around the outline, deep enough to cut through the rind, but avoid cutting into the flesh of the fruit.

-Gently lift out the center of the cervical model to expose the fruit beneath.

-Shave off the edge of the cervix to create the desired effacement.

-Record the cervical dilation and effacement on the answer key.

**Figure 4:**
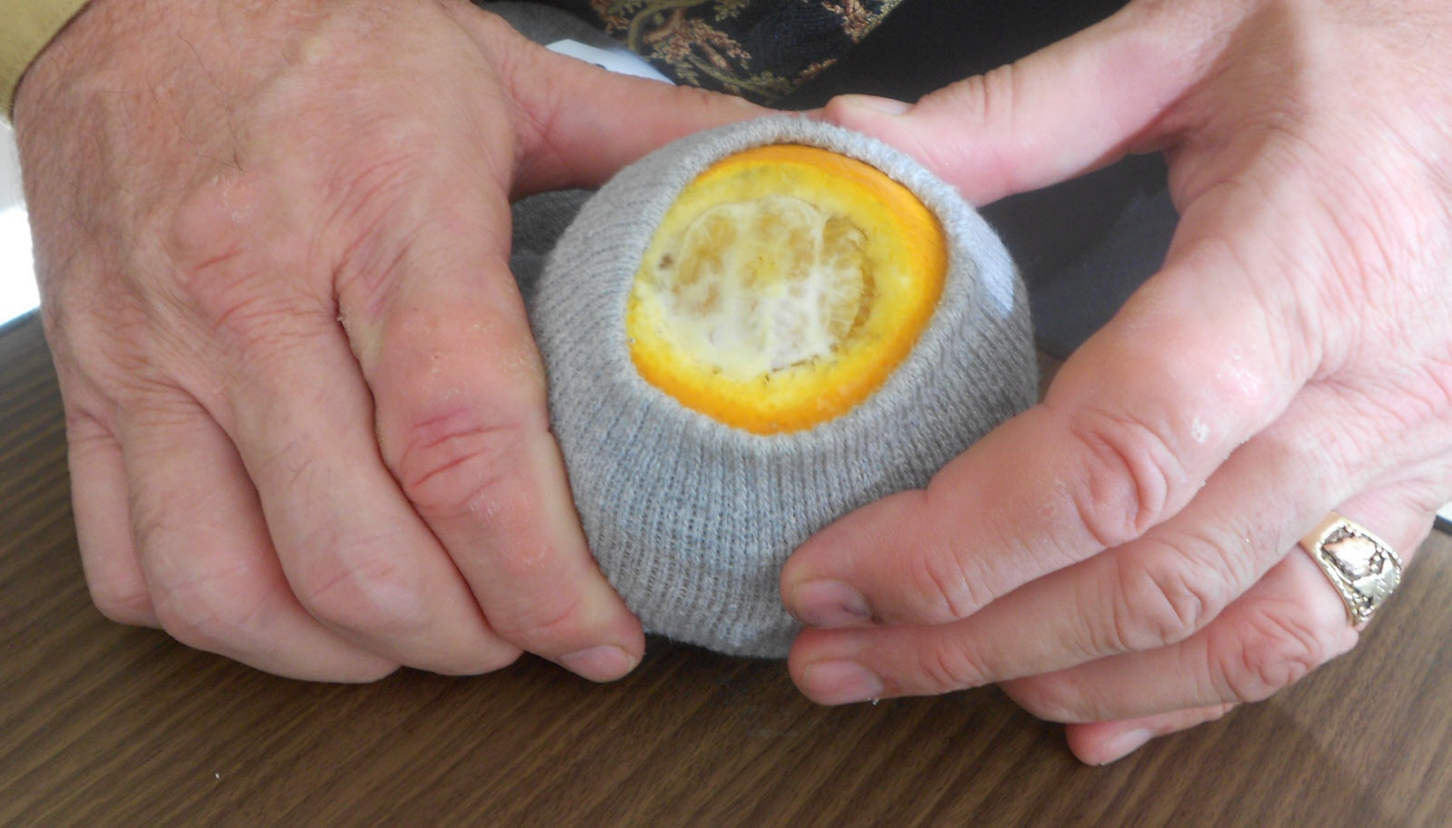
Positioning the cervical model such that it can be accessed through the neck of the sock. Once positioned, slide the sock up over the fruit, maintaining equal movement on all sides so that the "cervix" remains at the end of the neck.

-Insert the fruit into tube socks with the simulated cervix pointing up toward the neck of the sock (Figure 4).

-Roll the neck of the sock down to simulate the vaginal vault, making certain the fruit is not visible. A “vaginal vault” of 7.5 to 10 cm (3-4 inches) will suffice.

**Figure 5:**
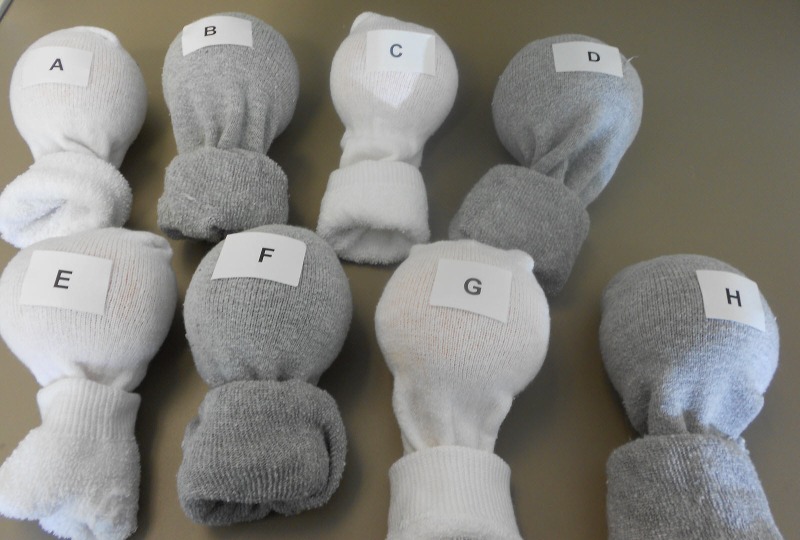
Models labeled and ready to be used

-Label each model according to the answer key (Figure 5).

-Untape the felt pen from the compass.

-Return the pencil tip to the compass for storage.

-Safely clean and store your knife and compass.

### Post-exercise cleanup

-Discard the fruit.

-Wash the socks.

-Store clean socks in a shoebox or other appropriate storage container along with the compass, the knife, the template, and a master copy of the recording sheet.

### Simulation exercise

**Figure 6:**
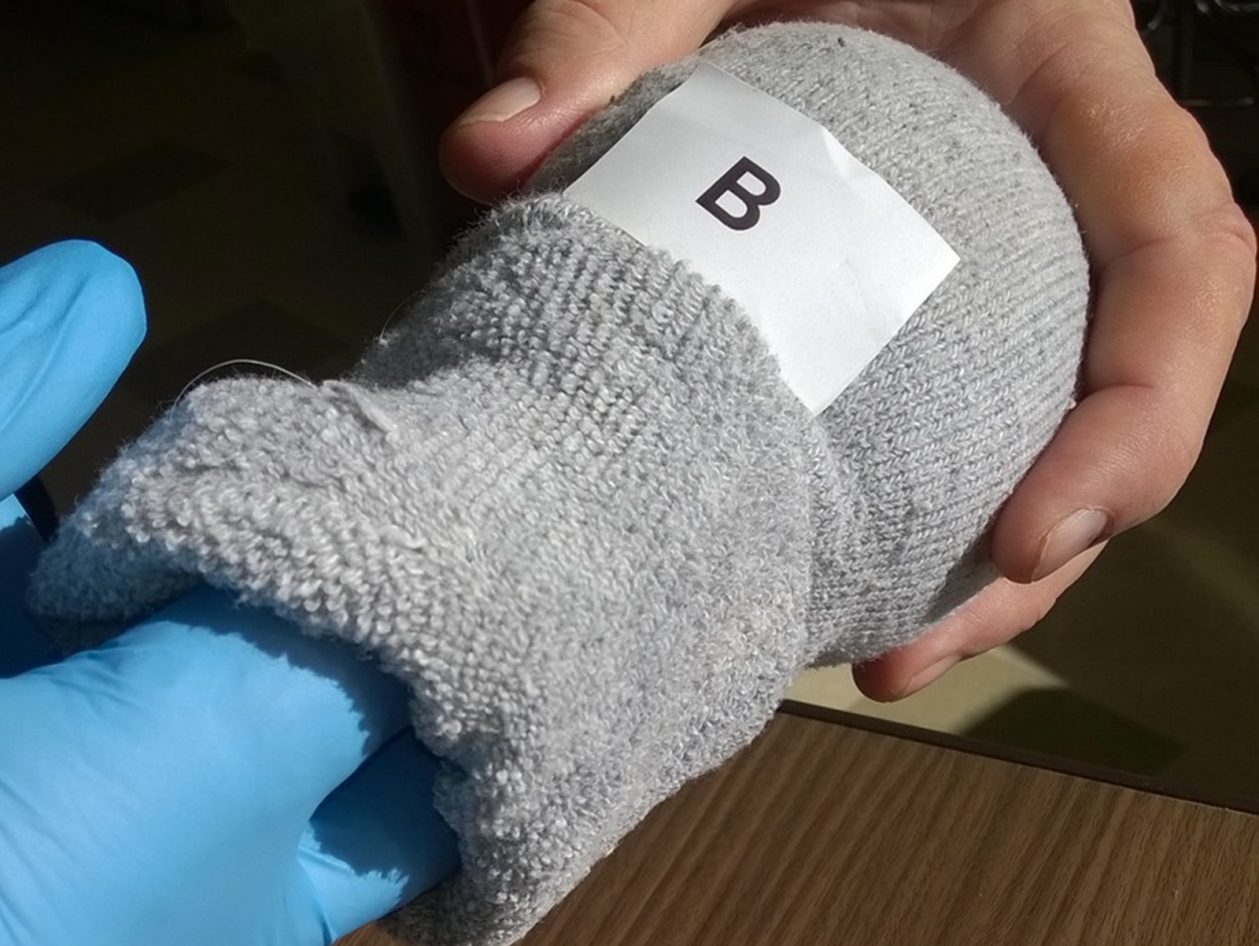
Simulated vaginal examination being performed by a student

Set out your models on a clean surface or surfaces. Supply learners with gloves and lubricant. Give each learner a recording sheet (Table 1). Every learner does a “vaginal exam” on each model and records their findings (Figure 6). When all learners have completed their score sheet, the answers are revealed. Learners with recorded findings most closely matching the correct answers “win” a prize. A discussion/debriefing completes the exercise.

## Discussion

This method of simulating cervical dilation and effacement provided a reasonably accurate model that gave students the opportunity to practice tactile skills, which are not routinely available to pre-licensure nursing students. The model was used both as a stand-alone simulation experience and as a “vignette” in a multi-station simulation activity. In all cases where the authors utilized the model, it generated valuable discussion that added richness to the debriefing.

The authors noted two limitations to this model. First, the thickness of the citrus fruit rind is not the actual length of a woman’s cervical canal. For example, the cervix at 0% effacement is 2 cm in length. Similarly, the cervix at 50% effacement measures approximately 1 cm in length. Since the typical grapefruit rind is approximately 1 cm in length, students must be informed that the citrus fruit examination model allows them to palpate a *relative* effacement of the cervix. Second, the model did not provide a method to simulate the station or the presentation of the fetus, which are key components in a complete vaginal examination and assessment of labor progression.

While the authors have described a cost-effective method of demonstrating relative cervical dilation and effacement, a systematic comparison of the citrus fruit model to commercially available cervical task trainers provides opportunities for future research.

## Conclusions

With few clinical sites allowing pre-licensure students to perform vaginal examinations during labor, most nursing students do not have an opportunity to practice the related tactile skills prior to obtaining their RN license. This simple, inexpensive simulation provides students with much needed practice building their manual assessment skills while performing the vaginal examination, but it also helps build their confidence as a practitioner in a safe and non-threatening environment.
